# Medical Aspects of Flying

**Published:** 1918

**Authors:** 


### AVIATION

**Medical Aspects of Flying.** Lieut.-Colonel James L. Birley, R. A. M. C., read a paper in which he stated that to be a successful pilot or observer a man must above all things possess " guts "; he must further have the flying temperament, and he must also be healthy. A man's past and family histories, his general demeanor and bearing are the best guides in assessing the stability of his nervous system and its capacity to withstand the various traumata to which it is going to be subjected. Pilots should come to their work fresh and keen, and it is wise to reject those men whose reserves of nervous energy have been partially exhausted by a long spell of service on the ground.

As regards vision, the British standards are comparatively low, as a man may wear glasses in the air and be a good pilot. Candidates must, however, have both color and stereoscopic vision.

The vestibular apparatus must be free from disease, and tests to confirm this should be made. In using the rotation tests the degree and duration of the forced movements induced, and also the degree and duration of the subjective sensations, are taken as guides rather than the nystagmus, which the British examiners consider too uncertain and too variable. The less the forced movement and subjective sensation the more likely is it, in their opinion, that the individual will not be unduly affected by the movements of the machine. If turning in the chair makes him feel sick and faint it is reasonable to suppose that spinning in the air will do the same. As regards night flying or flying in clouds a good deal of misapprehension seems to exist, and it has been stated that when deprived of sight the only impressions by which a man can be informed of his position relative to the earth are those arising from the labyrinth; whereas night flying pilots are unanimous in stating that they fly essentially by sight, using for this purpose an horizon of some sort or another. If they run into a thick fog, or are blinded by a flare or search-light, they do not know precisely where they are; and in the same way pilots have come out of a cloud in daylight upside down. Investigations show that the most common condition interfering with the functions of the labyrinth is obstruction of the Eustachian tubes, and the patency of both tubes should be insisted on in every candidate. The presence or absence of sea-sickness cannot be taken as necessarily indicative of a disordered labyrinth.

The general conclusions so far reached concerning the *period of inexperience* are briefly :

1. The labyrinth must be organically sound.

2. A normal labyrinth, deprived of the assistance of visual and muscle sense impressions, is not in itself sufficient to keep a pilot correctly informed of his position in space.

3. A labyrinth which over-reacts — or perhaps, more correctly a nervous system in which labyrinthine impressions are prone to radiate widely — is productive of ineffectiveness in the air.

In the second period, or *Period of Maximal Effectiveness*, attention is directed to the following points:

1. The abuse of alcohol must be strictly avoided.

2. Colds in the head and catarrhal conditions of the upper respiratory passages are apt to lead to Eustachian obstruction. They may also produce obstruction of the outlets from the frontal and sphenoidal sinuses, and if this occurs severe pain may be experienced at big altitudes owing to the fact that the imprisoned air is under greater pressure than the external air.

3. Due attention must be paid even to slight fevers, since they are prone to produce a temporary loss of tone in the cardiovascular and muscular systems.

4. All flying personnel should be warned of the importance of frequently opening the Eustachian tubes when flying and instructed as to how this can best be done. All cases of earache should be reported at once. Unilateral earache in pilots is nearly always the result of vascular congestion or even hemorrhage, secondary to Eustachian obstruction.

5. The clothing of aviators is of very great importance.

6. Crashes. The safety belt should encircle the chest rather than the abdomen, thereby minimizing the risk of the head falling forward and sustaining injury. The leather cushions on which the pilot sits must be fixed firmly to the seat. Due regard must be paid to crashes, however slight they appear to be.

7. The use of oxygen at high altitudes prolongs the period of maximal effectiveness and renders a man fully capable to discharge his duties in the air. The symptoms of oxygen want as they affect a man in the air are dyspnea, muscular weakness, impairment of judgment and *moral*, fainting or loss of consciousness, headache, over-filling of the bladder, vomiting. The symptoms noticeable after landing are:

(*a*) Fatigue. One has only to watch a flight landing after a two or three hours patrol without oxygen at 18,000 feet to realise the enormous strain which modern flying under active service conditions imposes. The difference when oxygen has been used is quite remarkable and highly encouraging.

(*b*) Frontal headache. This may be very severe indeed and persist for a whole day.

(*c*) Cardio-vascular system. The pulse is rapid and the diastolic pressure low, the result being that the pulse pressure is increased. In bad cases, however, the systolic pressure is also depressed and readings as low as 90 mm. Hg may be obtained.

(*d*) Respiratory system. Shortness of breath, inability to hold the breath, and even Cheyne-Stokes breathing, may persist for some time after landing.

*Diagnosis of oxygen want.* Dreyer's method is used, which consists in diluting the oxygen in the air breathed by the addition of nitrogen. It has been demonstrated that the normal individual reacts to the stimulus of lack of oxygen by all or some of the following changes: rise in pulse rate, rise of pulse pressure due to fall of diastolic pressure, deepening of the respiration, and concentration of the blood at expense of the plasma.

The third period, or *Period of Fatigue or Distress*, cannot be entirely anticipated. Its onset is subtle and depends on such variable factors as the individual, the type of work, the weather, enemy activity, etc. The results of exhaustion are summed up in three words: loss of effectiveness. The mental condition of a pilot in whom exhaustion is fully developed is highly characteristic. He becomes unsociable and irritable, is restless, can settle down to nothing, not even to write a letter or read a book, noise upsets him, he may hate hearing "shop" talked, and in many cases he cannot bear to see a machine landing on the aerodrome. His dash and confidence as a service pilot have disappeared, and his capabilities for flying have probably deteriorated, as evidenced by a series of bad landings and minor mishaps. A man who has reached this advanced stage will recover to a certain extent if rested, but for purposes of service flying he will probably never be the same man again.

The length of the period of stress should be limited in such a way that the rested man returns to the period of maximal effectiveness; and the flying personnel should be worked in short shifts, taking into consideration the nature of the work. The Commanding Officer and Medical Officer can do a great deal by working in close collaboration. The latter must not only be observant and approachable but must also possess a personality of his own and the gift of getting into close touch with his fellow men. Success in this part of his work can only be acquired by living amongst aviators and sharing their thoughts.

The paper read by Major FLACH has not been received. The paper on Gas Intoxication for obvious reasons cannot be published.

